# Stimulators of Mineralization Limit the Invasive Phenotype of Human Osteosarcoma Cells by a Mechanism Involving Impaired Invadopodia Formation

**DOI:** 10.1371/journal.pone.0109938

**Published:** 2014-10-14

**Authors:** Anna Cmoch, Paulina Podszywalow-Bartnicka, Malgorzata Palczewska, Katarzyna Piwocka, Patrick Groves, Slawomir Pikula

**Affiliations:** 1 Department of Biochemistry, Nencki Institute of Experimental Biology, Polish Academy of Sciences, Warsaw, Poland; 2 Laboratory of Cytometry, Nencki Institute of Experimental Biology, Polish Academy of Sciences, Warsaw, Poland; 3 Department of Biological Chemistry, Instituto de Tecnologia Quimica e Biologica, Universidade Nova de Lisboa, Oeiras, Portugal; Casey Eye Institute, United States of America

## Abstract

**Background:**

Osteosarcoma (OS) is a highly aggressive bone cancer affecting children and young adults. Growing evidence connects the invasive potential of OS cells with their ability to form invadopodia (structures specialized in extracellular matrix proteolysis).

**Results:**

In this study, we tested the hypothesis that commonly used *in vitro* stimulators of mineralization limit the invadopodia formation in OS cells. Here we examined the invasive potential of human osteoblast-like cells (Saos-2) and osteolytic-like (143B) OS cells treated with the stimulators of mineralization (ascorbic acid and B-glycerophosphate) and observed a significant difference in response of the tested cells to the treatment. In contrast to 143B cells, osteoblast-like cells developed a mineralization phenotype that was accompanied by a decreased proliferation rate, prolongation of the cell cycle progression and apoptosis. On the other hand, stimulators of mineralization limited osteolytic-like OS cell invasiveness into collagen matrix. We are the first to evidence the ability of 143B cells to degrade extracellular matrix to be driven by invadopodia. Herein, we show that this ability of osteolytic-like cells *in vitro* is limited by stimulators of mineralization.

**Conclusions:**

Our study demonstrates that mineralization competency determines the invasive potential of cancer cells. A better understanding of the molecular mechanisms by which stimulators of mineralization regulate and execute invadopodia formation would reveal novel clinical targets for treating osteosarcoma.

## Introduction

Osteosarcoma (OS) is an aggressive, drug-resistant cancer of bone with an unknown etiology and poor clinical outcome [1], [2]. Loss of control of cell proliferation and evasion from apoptosis appears to be a key mechanism in OS progression [3], [4], accompanied by high tendency for local invasion and early metastasis. It is established that cancer cell invasion requires changes in motility and degradation of the extracellular matrix (ECM). Secretion of enzymes modifying ECM is localized at specialized protrusions of cancer cells called invadopodia [5]. Invadopodia co-ordinate cell attachment to ECM with its degradation [6]. These protrusions facilitate migration and invasion due to their specific 3D actin organization and intense protein trafficking, which allow local delivery of integrins and proteolytic enzymes (metalloproteinases). Invadopodia are a key determinant in the malignant invasive progression of tumors [7] and nowadays represent an important target for cancer therapies [8]. Noteworthy, the marker protein of invadopodia, cortactin, has been recently confirmed as an enhancer of OS aggressiveness *in vivo* [9].

The accumulated evidence supports the notion that the osteogenic microenvironment could negatively contribute to osteosarcoma progression. It was reported that progression of OS and response to therapy is greatly influenced by the differentiation status of tumor cells [10]–[12]. Osteoblastic differentiation leads to acquisition of mineralization competence by the OS cells [13]–[16]. Additionally, recent reports have demonstrated that stimulators of mineralization *in vitro* (e.g. vitamin D [17], [18], P_i_ [19] or ascorbic acid [20]) suppress OS growth by inducing apoptosis. Furthermore, overexpression of proteins which contribute to the initiation of bone formation by driving osteoblastic differentiation reduced the metastatic potential of OS cells [21], [22].

Taken together, a possibility exists that the invasive potential of OS cells could be balanced by induction of mineralization. This prompted us to investigate the effects of stimulators of mineralization (ascorbic acid, B-glycerophosphate; AA/B-GP) on the invasive potential of OS cells. For this purpose, we characterized the response of human osteosarcoma cell lines, osteoblast-like Saos-2 cells [13], [14] and osteolytic-like 143B cells [15], [16], to treatment with AA/B-GP. We found that the effect of AA/B-GP depends on the ability of the OS cell line to mineralize ECM. This confirmed earlier observation that OS cells of osteoblastic phenotype are not invasive in contrast to highly invasive osteolytic-like cells [12], [23], [24]. In response to the treatment, osteoblast-like Saos-2 cells exhibited reduced proliferation rate and enhanced apoptosis, whilst the growth of osteolytic-like 143B cells was not affected. However, the invasive potential of 143B cells was significantly reduced in the presence of AA/B-GP. Here we identified invadopodia formation and matrix degradation as the critical invasion step that is affected by AA/B-GP.

## Materials and Methods

### Cells and treatment

Human osteosarcoma Saos-2 cells (American Type Culture Collection, ATCC No.:HTB-85) were cultured in McCoy’s 5A (PAA GE Healthcare, UK, Amersham Place) supplemented with 100 U/ml penicillin, 100 µg/ml streptomycin (Sigma Aldrich, USA, St. Louis) and 15% FBS (Fetal Bovine Serum, v/v, Gibco GE Healthcare). Human osteosarcoma 143B cells (American Type Culture Collection, ATCC CRL-8303) were cultured in Dulbecco’s Modified Eagle’s medium (4.5 g glucose/l, PAA GE Healthcare) supplemented with 100 U/ml penicillin, 100 µg/ml streptomycin (Sigma Aldrich) and 10% FBS (v/v, Gibco GE Healthcare). Cells were grown for 7 days (unless stated otherwise) under standard conditions (37°C, 5% CO_2_) in growth medium supplemented with 50 µg/ml ascorbic acid and 7.5 mM B-glycerophosphate (AA/B-GP; Sigma Aldrich) to stimulate mineralization [13], [14], [25], [26]. The culture media were changed every other day. Only cells between passages 2 and 9 were used in the experiments. Matrix mineralization was detected by Alizarin red S and von Kossa silver nitrate stainings which detect calcium and phosphate, as previously described [27], [28].

### Total cell lysate preparation and immunoblotting analysis

Cells were harvested and washed with phosphate buffered saline (PBS), pH 7.4. Cells were lysed with an ice-cold buffer containing 150 mM NaCl, 1% NP-40, 0.5% sodium deoxycholate, 0.1% SDS, 50 mM Tris pH 8.0, 10 mM NaF, 2 mM Na_3_VO_4_ and protein inhibitor cocktail (PIC; Sigma Aldrich), and then passed several times through a 26-gauge needle. The samples were centrifuged for 5 min at 800×g at 4°C. Protein concentration in the supernatant was determined using the Bradford method (BioRad Laboratories, USA, Hercules). Protein samples were diluted in Laemmli loading buffer and incubated at 100°C for 2 min. Total cell lysates (20 µg of protein) were separated by SDS-PAGE and transferred onto nitrocellulose membranes (Mini-PROTEAN III, BioRad Laboratories). After blocking with 5% low fat milk in TBS (Tris-buffered saline: 100 mM NaCl, 10 mM Tris pH 7.4), proteins were immunostained overnight with primary antibodies (**Table 1**) in 2.5% low fat milk in TBST (TBS with 0.05% Tween-20). The membrane was then incubated with horseradish peroxidase (HRP)-conjugated secondary antibodies (ECL-anti-mouse IgG-HRP or ECL-anti-rabbit IgG-HRP, both from BD Amersham Biosciences, GE Healthcare). The proteins were visualized by ECL kit according to the manufacturer’s instructions. β-Actin was used as an internal control.

**Table 1:** Primary antibodies used in the study.

antigen	gel band size (kDa)detected	host	supplier	dilution
Calcium sensing receptor	121		Abcam,Cambridge,USA	1∶500
Gelsolin	90	Rabbit		1∶1000
Cortactin	80	Mouse		1∶2500
Bone alkaline phosphatase[EPR4477]	58	Rabbit		1∶1000
BMP-2	44	Rabbit		1∶1000
β-actin	42	Mouse		1∶5000

### Alkaline phosphatase activity assay and mineral nodules staining

The alkaline phosphatase activity of cells was determined using freshly prepared 10 mM p-NPP (4-nitrophenyl phosphate, disodium salt, hexahydrate) in reaction buffer (25 mM glycine, 25 mM piperazine, pH 10.4). The reaction was started by addition of freshly obtained cell lysates to the reaction buffer and incubation at 37°C. The absorbance was measured at 30 sec intervals at 420 nm in a SpectraMax M5e Microplate Reader (Molecular Devices, USA, Sunnyvale). The alkaline phosphatase activity was quantified using a molar absorption coefficient of 17,800 cm^−1^ M^−1^ The enzyme activity was expressed in µmoles of p-NPP hydrolyzed per minute per milligram of total protein. Total protein content in cell lysates was measured by the Bradford assay. At least 6 samples were analyzed in each experimental condition.

### Proliferation assay

Cells were labeled with 10 µM cell proliferation dye eFluor 670 (eBioscience, USA, San Diego) according to the manufacture’s protocol and then cultured for 7 days in control or AA/B-GP supplemented medium. After detachment with trypsin solution and washing in PBS pH 7.4, fluorescence intensity of the cells was measured by flow cytometry (FACSCalibur, Becton Dickinson, USA, San Jose) and analyzed with CellQuestPro software (Becton Dickinson).

### Cell cycle analysis

Cell cycle analysis was performed according to the method of Pozarowski and Darzynkiewicz [29]. Cells were detached with trypsin solution from the culture flask, subsequently washed and suspended in 200 µL of PBS. Then, the cells were fixed with ice-cold 70% ethanol overnight at −20°C. After washing in PBS, the cells were incubated for 5 min at RT in extraction buffer consisting of 4 µM citric acid in 0.2 M Na_2_HPO_4_ followed by DNA staining at 37°C for 30 min in a buffer consisting of 3.8 mM sodium citrate, 50 µg/ml 4′,6-diamidino-2-phenylindole (DAPI), 0.005 µg/ml RNAse A. DNA content was measured using a BD LSRFortessa flow cytometer (Becton Dickinson) and analyzed using the ModFit LT software (Verity Software House, USA, Inc, Topsham).

### Annexin V and caspase flow cytometry analysis

Control or 7 day AA/BGP treated cells were harvested with StemPro Accutase Cell Dissociation Reagent (Gibco GE Healthcare). Apoptosis analysis was performed using the PE Annexin-V/7-AAD Apoptosis Detection Kit (BD Biosciences Pharmingen) or multicaspase fluorogenic substrate (SR) (Guava-Merck Millipore, USA, Billerica) according to the manufacturer’s instructions. Briefly, cells were washed in PBS and suspended in binding buffer for staining with PE Annexin-V and 7-AAD (7-aminoactinomycin D) at room temperature for 15 min in the dark. The cells were analyzed by flow cytometry (FACSCalibur, Becton Dickinson). The signal obtained from cells stained with annexin-V or 7-AAD alone was used for fluorescence compensation. To measure caspase activation the cells were incubated with the multicaspase substrate for 1 h under cell culture conditions followed by staining with 7-AAD. Fluorescence was determined using the microplate reader in a Guava easyCyte 8HT Benchtop Flow Cytometer (Guava-Merck Millipore) and acquired using the Guava Caspase Software Module. Cells stained with SR or 7-AAD alone were used for fluorescence compensation.

### *In* *vitro* migration assay

For wound healing experiments, cells were seeded on 12-well plates to reach 80% confluence at the day of wounding. Cells were wounded by the tip of a micropipette and washed with PBS pH 7.4 to remove floating cells. Then, cells were fed with fresh medium. Cell movement was followed for a period of 48 h. Time-lapse observations were performed using Leica AF7000 Live Imaging System (Leica Microsystems GmbH, Germany, Wetzlar) microscope with environmental chamber, objective 10x/0.40 numerical aperture (NA). Wound closure was calculated and expressed as a percentage of the area of the initial wound (defined at 0 time point). Image analysis was performed using the NIH ImageJ software (National Institutes of Health, USA, Bethesda).

### Transwell invasion in collagen matrix

Cell invasion was examined using inserts with polycarbonate filters (8 µm pore size; BD Biosciences GE Healthcare). The upper side of the polycarbonate filter was either not coated or coated with 0.5 mg/ml collagen I (Life Technologies, USA, Carlsbad) for 4 h to form a continuous thin layer. After 7 days of treatment, cells were seeded into the upper chamber in serum free medium in the presence or absence of AA/B-GP. The lower chamber was filled with complete medium. After 20 h of incubation, the cells in the upper chamber of the filter were removed with a cotton swab. Invasive cells on the underside were stained with DAPI and counted under the Leica fluorescent microscope DMI6000 objective 10x/0.25 (Leica Microsystems) in 16 random fields. The number of cells was determined with NIH ImageJ software. The invasion index is expressed as percentage of invading cells over the total cell input.

### Adhesion assay and crystal violet staining

24-well plates were coated with collagen type I at 10 µg/ml and blocked with 0.1% bovine serum albumin (BSA) in PBS pH 7.4 for 1 h. Then 3.0×10^5^ cells per well were incubated for different periods of time (5, 15 and 25 min) under control conditions or in the presence of AA/B-GP. After removal of not adhering cells by PBS washing the remaining cells were fixed with 3.7% (w/v) PFA (paraformaldehyde) for 15 min and stained with 0.5% crystal violet in 25% methanol for 2 h. This was followed by extensive washing with double distilled H_2_O and drying overnight. The attached cells were observed using an inverted light microscope (Zeiss Axio Observer, Germany, Oberkochen). Next, crystal violet was dissolved in 10% acetic acid and absorbance at 590 nm was measured in a scanning multi-well spectrophotometer SpectraMax M5e Microplate Reader (Molecular Devices).

### Visualization of invadopodia

Cells were seeded on glass cover slips coated with collagen I at 10 µg/ml and allowed to adhere at 37°C in a 5% CO_2_ humidified atmosphere for 20 h. Cells were washed with PD buffer (125 mM NaCl, 5 mM KCl, 10 mM NaHCO_3_, 1 mM KH_2_PO_4_, 10 mM glucose, 20 mM HEPES, pH 6.9) and fixed with 3.7% (w/v) PFA in PD buffer. Fixed cells were incubated in 50 mM NH_4_Cl in PD buffer and then permeabilized with 0.08% Triton X-100 in PD buffer (5 min, 4°C). After additional washing with PD buffer and TBS, cells were incubated for 1 h with a blocking solution, 5% FBS in TBS. Then, the slides were incubated with anti-cortactin antibody and anti-gelsolin antibody (**Table 1**), diluted in TBS containing 0.5% FBS and 0.05% Tween-20. After 1.5 h of incubation, cells were washed and then incubated for 1 h with goat anti-mouse IgG–fluorescein isothiocyanate (FITC, 1∶200 v/v) and anti-rabbit tetramethylrhodamine (TRITC, 1∶200 v/v) (Sigma Aldrich). After washing, cells were mounted in 0.6% Moviol 4–88/2.5% DABCO resin (Sigma Aldrich). The specimens were studied with a confocal microscope Leica SP8 (Leica Microsystems) using a 63×/numerical aperture (NA) 1.4 Plan-Neofluar objective. To prevent overlapping of the fluorescence signal emitted by fluorochromes, each channel was imaged sequentially using the multitrack recording module before merging. Z-stack pictures were obtained every 0.2 µm using the LAS AF software (Leica Microsystems GmbH). Selected image stacks were further subjected to deconvolution (Huygens Software, Scientific Volume Imaging, the Netherlands, Hilversum). Three-dimensional reconstructions were obtained by using Imaris software (Bitplane, Switzerland, Zurich). For the measurements, at least 15 cells were analyzed per condition across two independent experiments.

### Fluorescent gelatin degradation assay

The matrix degradation assay was conducted as described previously [30]. Glass-bottom collagen I coated dishes (35 mm, MatTek Corporation, USA, Ashland) were further coated with Oregon green 488 gelatin (Life Technologies) according to the manufacturer’s protocol. Next, 3×10^4^ cells were seeded on each plate and cultured O/N. Cells were fixed with 3.7% PFA, permeabilized with 0.05% Triton-X-100, then blocked with 5% FBS and probed for F-actin (TRITC-phalloidin; Sigma Aldrich). Images of the cells were collected using a confocal microscope Leica SP8 using a 63×/NA 1.4 Plan-Neofluar objective. Invadopodia were manually counted as actin-positive dots associated with gelatin degradation. Matrix degradation areas were calculated as the total area covered by degradation holes/field in thresholded images using the Analyze Particles tool in the NIH ImageJ software and normalized to the cells area in each field.

### Statistical analysis

All data are presented as mean ± SEM of n observations. The experiments were conducted three times unless stated otherwise. Data were analyzed using Student’s t test at *p<0.05.

## Results

### The ability of OS cells to differentiate and mineralize in the presence of AA/B-GP

**Figure 1:**
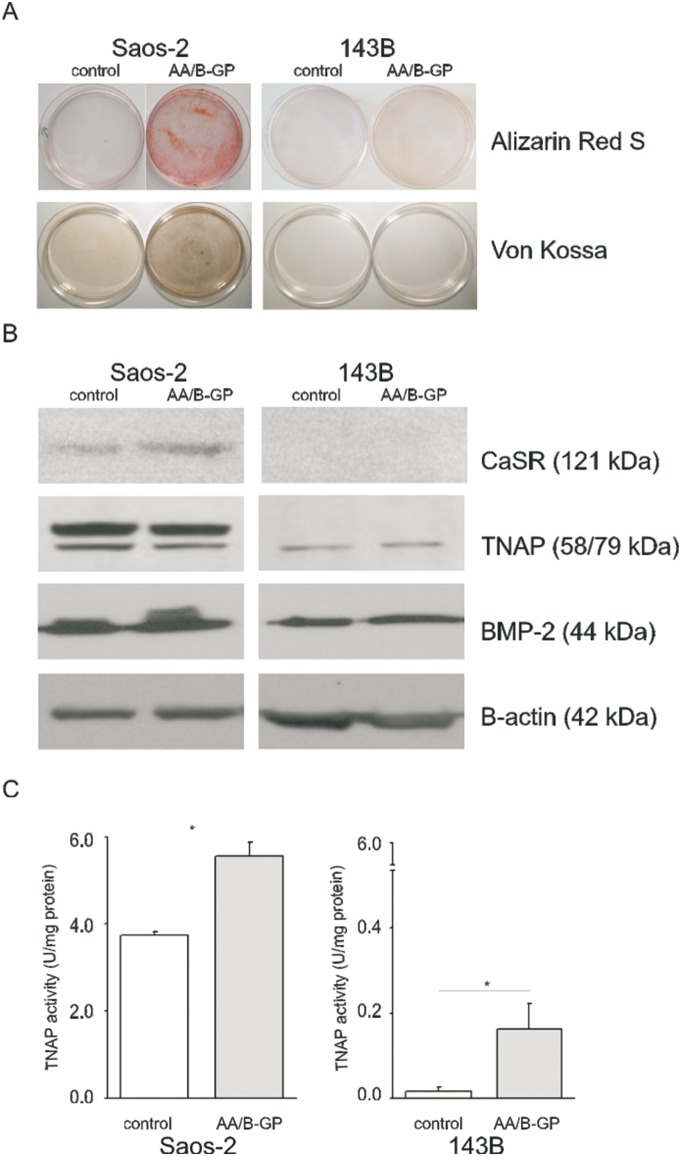
The effect of stimulation with ascorbic acid and β-glycerophosphate on the mineralization of Saos-2 and 143B cells *in* *vitro*. (A) Plate view of von Kossa and Alizarin red S stainings of mineral nodules formation in cultured cells after 7 days of stimulation with AA/B-GP. (B) Identification of osteogenic markers (TNAP, 58 kDa; CaSR, 121 kDa; BMP-2, 44 kDa) in whole cell lysates (25 µg protein/well). Protein content was analyzed by immunoblotting and standardized to the β-actin level. (C) Total TNAP activity was measured in cell lysates, normalized to the cellular protein content and presented in units (1 U = 1 µmole of p-NPP hydrolysed per minute) per milligram of protein. Error bars indicate means ± SEM; n = 5, *p<0.05 by Student’s t-test.

As a starting point of the study, we verified the osteoblastic versus osteolytic phenotype of the employed OS cell lines and their response to stimulation of mineralization with AA/B-GP. To this end we compared the cellular level of osteogenic differentiation markers. As shown in **Figure 1A**, the content of TNAP (tissue non-specific alkaline phosphatase), BMP-2 (bone morphogenetic protein 2) and CaSR (calcium sensing receptor) was drastically higher in Saos-2 OS cells. Treatment of OS cells with AA/B-GP for 7 days did not alter the pattern of the osteogenic markers (**Fig. 1A**) but resulted in a significant increase of TNAP cellular activity of Saos-2 cells (**Fig. 1B**). Contrary to Saos-2 cells, the TNAP activity measured in 143B cells was negligible (**Fig. 1B**). The obtained results demonstrate the difference between the two cell lines in terms of their competence to mineralize ECM.

### Mineralization of osteoblast-like OS cells is accompanied by growth inhibition and apoptosis

**Figure 2:**
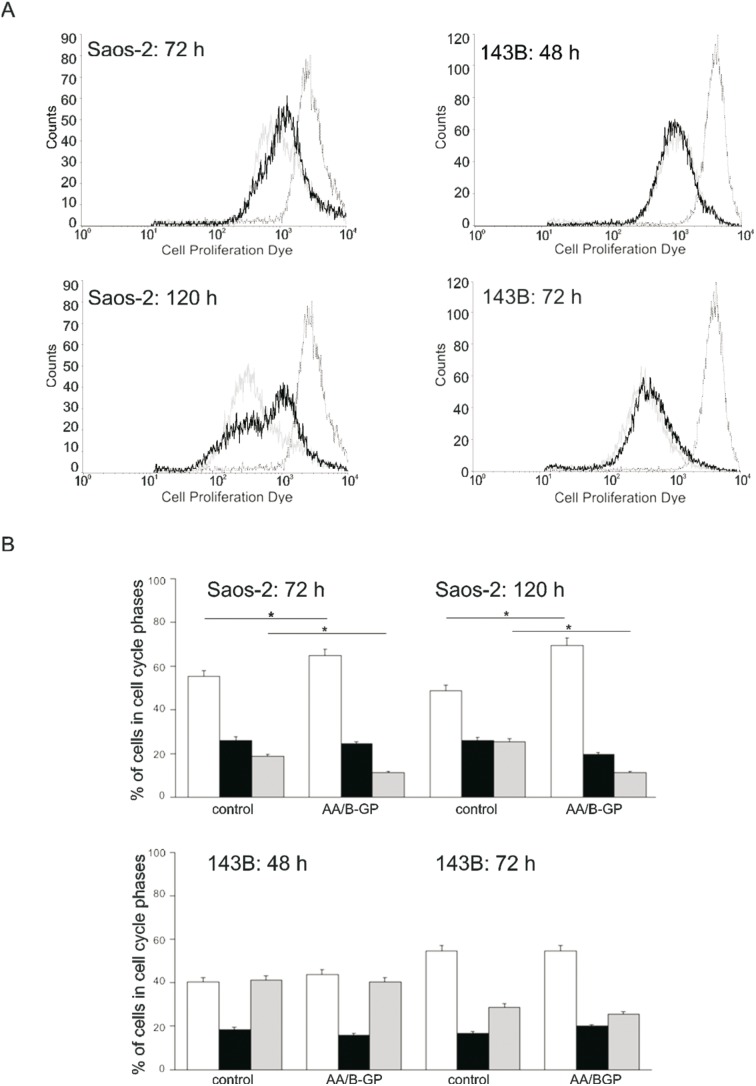
Growth of osteosarcoma Saos-2 and 143B cells treated with ascorbic acid and β-glycerophosphate. (A) Analysis of cell proliferation rate by flow cytometry. Cells were stained with Alexa Fluor 670. Representative plots of fluorescence measurements at the time points after the 1^st^ and 2^nd^ division of Saos-2 or 2^nd^ and 4^th^ division of 143B cell line. Histograms: dashed black line - time 0, continuous gray line - control, continuous black line - AA/B-GP treatment. (B) Determination of cell cycle progression in osteosarcoma cells. Representative plots showing analysis at the time points after the 1^st^ and 2^nd^ division of Saos-2 or 2^nd^ and 4^th^ division of 143B cell line. Percentages of cells in the G0/G1, S, and G2–M phases are presented for each experimental group. Bars represent: open-G0/G1 phases, filled -S phase, gray – G2/M phase. Error bars indicate means ± SEM; n = 3, *p≤0.05 by Student’s t-test.

In order to verify the effect of AA/B-GP on the proliferation and viability of OS cells the flow cytometry methods were employed. For proliferation analysis (**Fig. 2A**) and cell cycle distribution (**Fig. 2B**), cells were assessed for analysis every 24 h until the 7^th^ day of the experiment. Given the difference in the duration of the cell cycle between the two cell lines the time points corresponding to the completion of the 1^st^ and 2^nd^ division or 2^nd^ and 4^th^ division were chosen to be 72 h and 120 h for Saos-2 cells and 48 h and 72 h for 143B cells, respectively. The effect of AA/B-GP on growth and viability occurred to be cell-type dependent for the tested OS lines. The AA/B-GP treatment resulted in a significant reduction of Saos-2 cell proliferation rate (**Fig. 2A**). The GeoMean values were higher in cells stimulated for mineralization by 28% (870 and 1121, for control and AA/BGP treated cells, respectively) at 72 h and by 50% after 120 h.

Next, to reveal the mechanism of the inhibitory effect of AA/B-GP on Saos-2 cell proliferation, the cell cycle distribution was examined (**Fig. 2B**). The percentage of cells in each cell cycle phase (G0/G1, G2/M and S) was assessed using flow cytometry analysis after DNA staining with DAPI [23]. **Figure 2B** depicts that after 72 h of treatment with AA/B-GP about 65% of the population of Saos-2 cells were arrested in the G0/G1 phase. Thus, upon stimulation to mineralization, about 10% more cells were in the G0/G1 phase than in the population of untreated cells. At 120 h the amount of cells in G0/G1 was increased to 80%.

**Figure 3:**
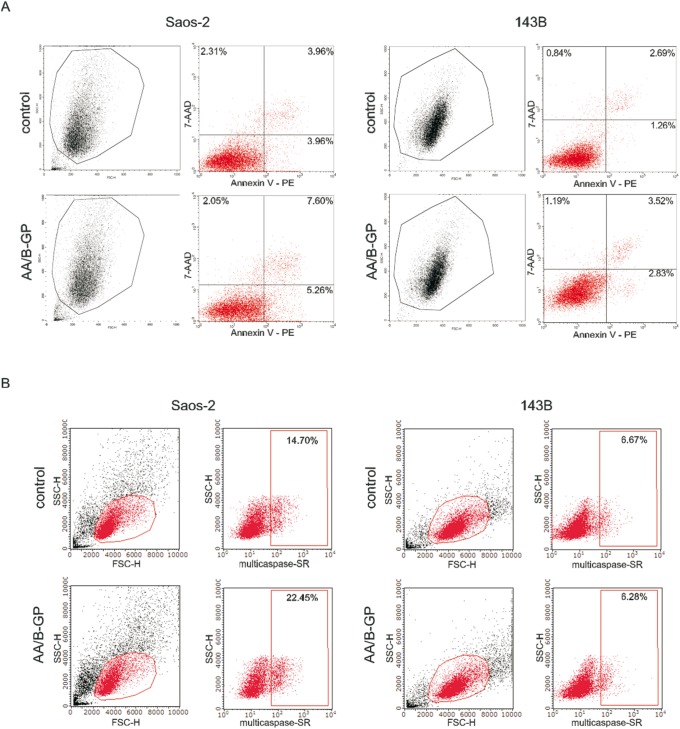
Viability of osteosarcoma Saos-2 and 143B cells treated with ascorbic acid and B-glycerophosphate. (A) Determination of apoptosis by Annexin-V assay. Cells after 7 days of stimulation with AA/B-GP were double stained with PE-Annexin-V/7-AAD. The cells gated through the FSC vs SSC plot were further analyzed for fluorescence intensity of PE-Annexin-V vs 7-AAD. The Annexin-V^+^7-AAD^−^ cells were considered as early apoptotic, while the Annexin-V^+^7-AAD^+^ cells were counted as late apoptotic. The percentage of each population is indicated on the graphs. Representative graphs are presented. (B) Analysis of multiple caspase activation and 7-AAD permeability in apoptotic cells. Cells were stained with SR-Peptide Fluor and 7-AAD after 7 days of stimulation with AA/B-GP. The cells gated through the FSC vs SSC dot plot were further analyzed for fluorescence intensity of the orange-red fluorescent probe (SR). Positively labeled cells are detected in the red square gate. The percentage of multicaspase positive cell population is indicated on the graphs. Data are representative of three independent experiments.

To further elucidate the effect of AA/B-GP on cell viability the degree of cell apoptosis was tested using the Annexin-V assay (**Fig. 3**). This assay allowed us to distinguish early apoptotic cells (annexin V positive only) from late apoptotic/necrotic cells (Annexin-V and 7AAD positive). Upon a 7 day treatment with AA/B-GP about 13% of the population of Saos-2 cells were positive for annexin V whilst in control conditions 8%. Altogether, the study demonstrated that the effect of AA/B-GP on Saos-2 cell growth was accompanied by ongoing apoptosis. This was additionally confirmed by measurement of multiple caspase activation (**Fig. 3B**) as a critical early step in the onset of apoptosis. For that purpose we employed fluorescent multicaspase reagents and the 7-AAD assay which allow to measure intracellular levels of the enzyme without using harsh lysis methods. In this experiment we distinguished caspase-positive (SR-Peptide-positive) population of live cells undergoing apoptosis. The population of caspase positive cells in control Saos-2 cells amounts to 14.7% while in AA/BGP-treated cells it is 22.4%. After 7 days of treatment with AA/BGP the caspase positive population of 143B cells (6.26%) is similar in size to that in control (6.7%). In summary, the flow cytometry analysis demonstrated an increase in the activity of multiple caspases in mineralizing Saos-2 cells compared to the control.

The treatment of 143B cells with AA/B-GP did not reveal any significant changes in their proliferation rate (**Fig. 2A**) or cell cycle distribution (**Fig. 2B**). The 72 h analysis of cell cycle distribution revealed similar percentages of cells in G0/G1 (54%), G2/M (16–19%) and S phase (28–25%) for control and AA/B-GP treated 143B cells. Noteworthy, after 7 days of stimulation with AA/B-GP, more than 93% of 143B cells were negative for Annexin-V (**Fig. 3, right panel**). Taken together, the two investigated OS cell lines differ with respect to their growth and viability upon treatment with stimulators of mineralization.

### Prolonged AA/B-GP treatment of OS cells limits their migration in *vitro*

**Figure 4:**
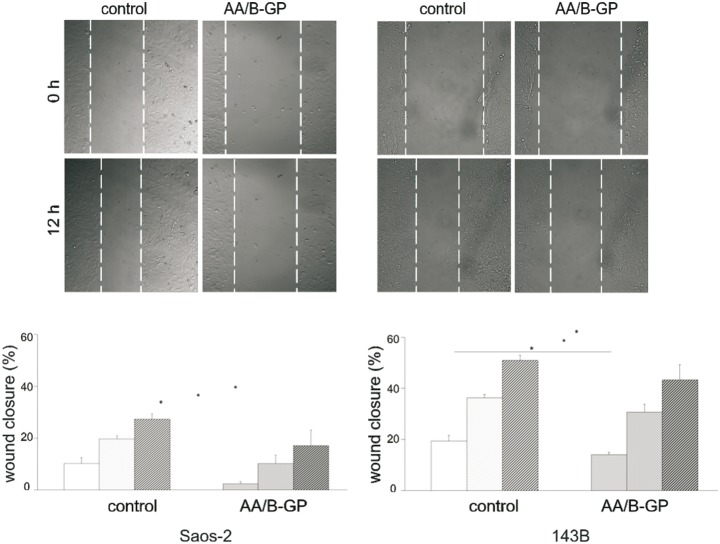
Migration of osteosarcoma Saos-2 and 143B cells in the presence of ascorbic acid and β-glycerophosphate. Uniform scratches were created in confluent cell cultures, which were treated with AA/B-GP over a period of 7 days. The upper panels show representative images of wound closure in control osteosarcoma cells and cells stimulated with AA/B-GP, 12 h after scratching. Wound borders are marked with dashed lines. The lower panels represent the quantification of wound closure in % 4 h, 8 h and 12 h after injury (time 0). Time lapse imaging of wound closure was captured using a Leica AF7000 microscope Live Imaging System at 10× objective. Bars represent: open – 4 h after injury; dotted – 8 h after injury; grey stripped – 12 h after injury. The mean value for three individual experiments ± SEM is shown. Error bars indicate means ± SEM; n = 6, *p≤0.05 by Student’s t-test.

Since cell migration is a prerequisite for tumor invasion and metastasis, we evaluated the influence of prolonged exposure of osteosarcoma cells to AA/B-GP on their migratory activities using a wound-healing assay. **Fig. 4** **(lower panels)** represents quantization of the wound closure (in %) 4, 8 and 12 h after the injury was inflicted in a 80% confluent cell monolayer, maintained in control or AA/B-GP supplemented medium for 7 days. The wound closure by Saos-2 cells after 4 h was 10.2%±2.1 in control and 2.3%±0.9 in AA/B-GP-treated cells, whilst after 12 h: 27.3%±1.9 and 17.19%±6.0, respectively. Irrespective of conditions wound closure by 143B cells was enhanced when compared to Saos-2 cells. In the case of 143B cells the wound closure 4 h post injury was 19.4%±1. 7 in control and 13.9%±1.3 in AA/B-GP-treated cells, while after 12 h it was 50.9%±2.9 and 43.3%±3.3, respectively. Therefore, regarding 143B cells, the effect of AA/B-GP on wound closure did not exceed 6% compared to control conditions. Taken together, stimulation to mineralization by the AA/B-GP treatment was accompanied by a reduced migration rate of cells of both OS cell lines.

### Mineralization stimulators elicit an inhibitory effect on the adhesion of 143B osteosarcoma cells to type I collagen

**Figure 5:**
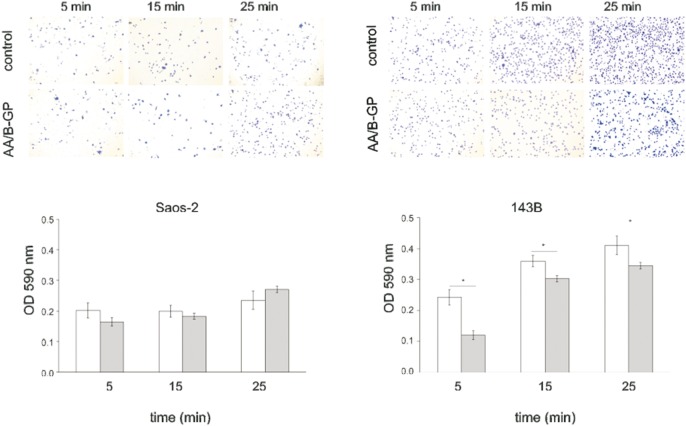
Comparison of the adhesive abilities of osteosarcoma cells treated with ascorbic acid and β-glycerophosphate. After 7 days of treatment cells were detached and incubated at 37°C for 5, 15, 25 min on 10 µg/ml of collagen type I matrix. Histogram of crystal violet absorbance at 590 nm and representative photographs (objective 10X) of stained adherent cells are shown. Data are expressed as means ± SEM from three independent experiments. Bars represent: open - control cells; filled- cells cultured in media supplemented with 50 µg/ml ascorbic acid and 7.5 mM β-glycerophosphate. Error bars indicate means ± SEM; n = 3, *p≤005 by Student’s t-test.

To further elucidate the possible mechanisms of migration inhibition in OS cells after treatment with AA/B-GP, we performed an adhesion assay on collagen type I (**Fig. 5**). Collagen type I was chosen for tests as the most abundant protein in the body and the structural scaffold upon which bone is built [31]. To this end the cells were preconditioned for 7 days with AA/B-GP and then seeded onto the collagen matrix (supplementation was preserved). Cellular adhesion at indicated time points was measured spectrometrically. **Figure 5** shows that Saos-2 cell adhesiveness was not modified by AA/B-GP at any of the time points investigated. Contrary to that, the adhesion ability of 143B cells was limited in the presence of AA/B-GP in a statistically significant way when compared to the control conditions. Five min after seeding in the presence of AA/B-GP, the OD_590__nm_ was 0.120±0.06 with respect to 0.410±0.01 for the control and, 25 min after seeding, the OD_590__nm_ in AA/B-GP-treated cells was 0.344±0.01 with respect to 0.410±0.01 for the control. In general, the ability of 143B cells to adhere to collagen matrix was visibly greater than that of Saos-2 cells.

### Sustained exposure to AA/B-GP results in reduced invasiveness of 143B cells

**Figure 6:**
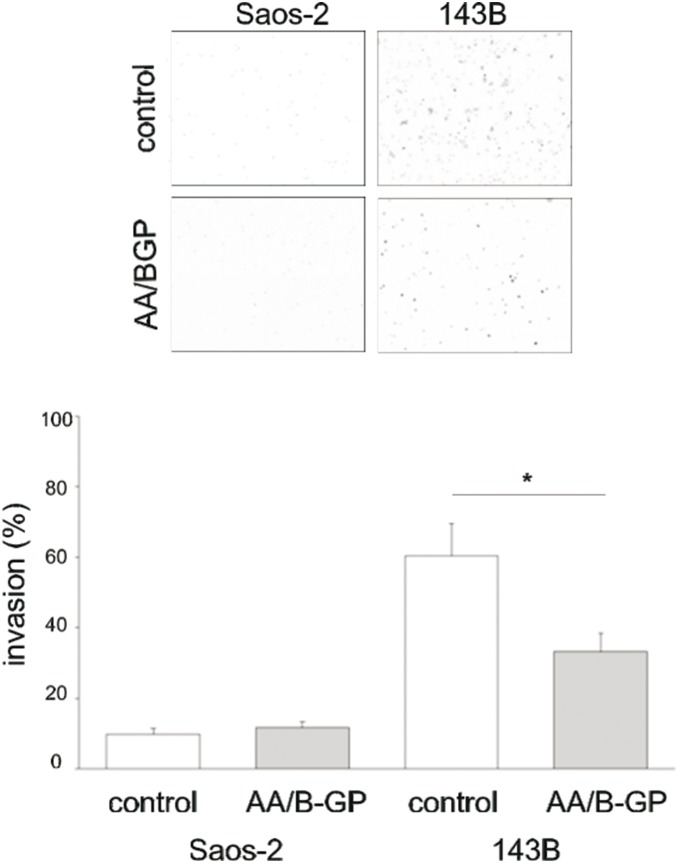
Invasiveness potency of cells treated with ascorbic acid and β-glycerophosphate. Cell invasiveness was assessed using transwells with collagen type I gel. Invasion through the membrane was determined for a time period of 20 h. Invading cells from 16 randomly chosen fields were counted for each transwell. Image analysis was performed using NIH ImageJ. Upper panel shows representative observation of invading cells and lower panel shows % invasion index (expressed as percentage of invading cells over the total cell input). Bars represent: open - control cells; filled- cells cultured in media supplemented with 50 µg/ml ascorbic acid and 7.5 mM β-glycerophosphate for 7 days. Data are expressed as means ± SEM from three experiments. Error bars indicate means ± SEM; n = 3, *p≤005 by Student’s t-test.

As the ability of cancer cells to adhere and interact with the different components of the extracellular matrix is essential for cell invasion, we employed a transwell assay to test the invasiveness of OS cells *in* *vitro* (**Fig. 6**). Cells were pre-cultured for 6 days under control or AA/B-GP treatment conditions and then seeded on the top of collagen gel in the transwell chamber and allowed to invade for 20 h. The osteoblast-like Saos-2 cells were almost non-invasive/essentially non-invasive (invasion index for control 9.0±2% and for AA/B-GP treated 11.7±2%). In contrast, the 143B cells appeared to be highly invasive (invasion index for control cells 60.3±2%). The obtained results indicated that the invasive potential of 143B cells was significantly reduced when cells were exposed to AA/B-GP (invasion index 33.3±3%). This observation has been additionally tested by the ability of the two types of OS cells to form invadopodia.

### Saos-2 and 143B cells exhibit different potency to form functional invadopodia

**Figure 7:**
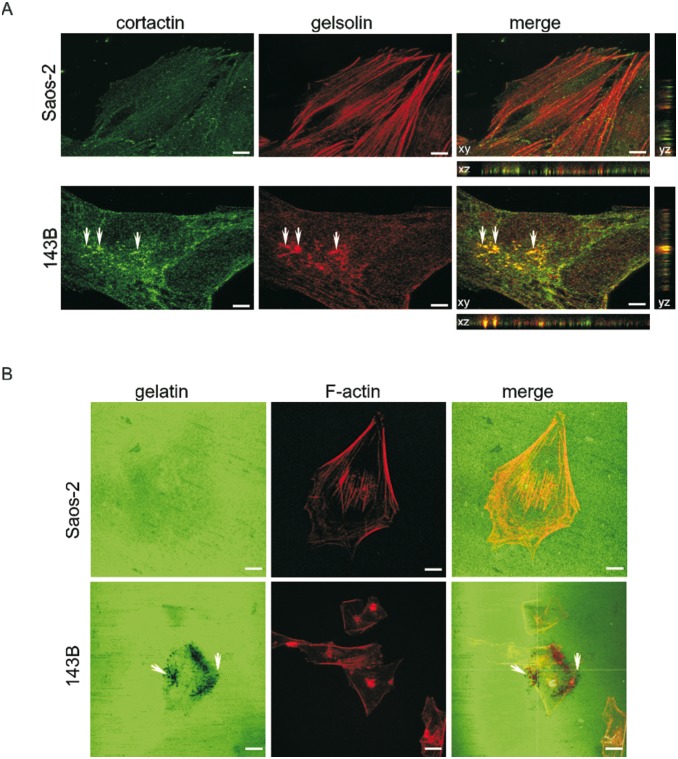
Comparison of osteosarcoma cell ability to form invadopodia. (A) Representative confocal microscopy images of proteins associated with invadopodia formation in OS cells. Cells stained for cortactin (green) and gelsolin (red). Invadopodia marked with arrowheads. Optical sections taken every 0.20 µm by a confocal microscope (Leica SP8, objective 63x/1.40 Oil). Reconstruction of the Z-cut section profile is shown. Scale bar = 20 µm. (B) Fluorescence analysis of the invadopodia activity in the matrix degradation assay. Representative images show oregon green-488 labeled gelatin (left panels) and phalloidin-TRITC stained actin (middle panels). Merged image (right panel) presents localization of F-actin dots within the degradation area (punctate areas devoid of staining). The x–y optical sections taken at 0.4 µm intervals by a confocal microscope (Leica SP8, objective 63x/1.40 Oil). Scale bar = 20 µm.

The results described above show that the osteoblast-like Saos-2 osteosarcoma cells competent to mineralization exhibit relatively low motility and invasiveness when compared to osteolytic-like 143B cells. Here, we have further investigated this phenomenon using the invadopodia formation assay. Invadopodia are persitent protrusions formed on the ventral side of cancer cells, essential for ECM degradation during invasion and metastasis. Here we imaged invadopodia formed by control OS cells under the confocal microscope using immuno-co-localization of marker proteins (cortactin and gelsolin) (**Fig. 7A**). Cortactin and gelsolin were observed to co-localize in numerous invadopodia formed by 143B cells. The Z-stack observations showed that these structures were approximately a micron in width and ranged from 3 to 5 µm in length. We found that invadopodia occurred mostly in 143B cells and were rarely found in Saos-2 cells.

To visualize the proteolytic activity of invadopodia, it was necessary to perform an additional study that combines high resolution imaging with *in* *situ* zymography. Therefore, we utilized the classic technique in which cells were plated on top of a chemically cross-linked layer of gelatin labeled with Oregon Green 488 and focal digestion was observed in time as the disappearance of the green substrate fluorescence (**Fig. 7B**). Before examination under the confocal microscope, cells were preserved and stained with TRITC-phalloidin to reveal the actin cytoskeleton. Actin dense cores were associated with areas of local gelatin degradation dots in 143B cells.

### AA/B-GP treatment affects invadopodia formation and proteolytic activity of 143B cells

**Figure 8:**
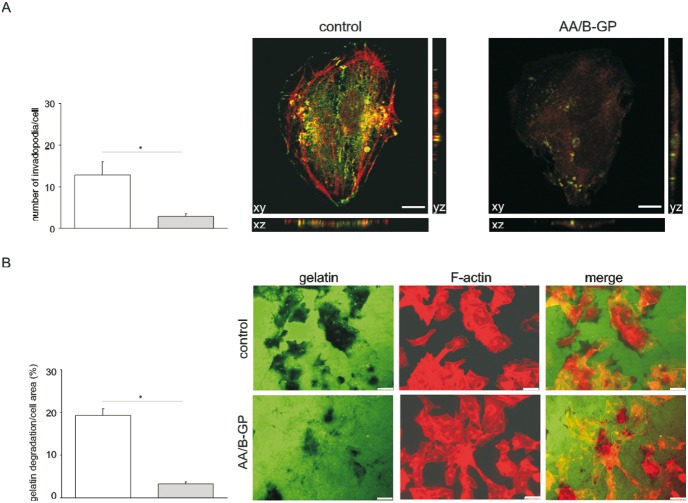
Invadopodia formation and proteolytic activity in 143B cells treated with ascorbic acid and β-glycerophosphate. 143B cells were cultured in control conditions or in the presence of AA/B-GP for 24 h. (A) Determination of the number of invadopodia per cell. Invadopodia of cells that had assembled at least one invadopodium were counted in 10 visual fields. Data are expressed as means values ± SEM from multiple experiments, *p≤0.05 by Student’s t-test. Panel on the left shows representative confocal images of invadopodia (counterstained for cortactin and F-actin) in 143B cells. Reconstruction of the Z-cut section profile is shown. Scale bar = 20 µm. (B) Analysis of fluorescent matrix degradation normalized to total area of the cells. Data are expressed as means ± SEM from multiple experiments, *p≤0.05 by Student’s t-test. Typical images of Oregon green-488 labeled gelatin (left panels), phalloidin-TRITC stained F-actin (middle panels) and merged images (right panel) are presented. Scale bar = 50 µm.

Finally, we addressed the question of the effect of AA/B-GP on the invasiveness of 143B cells through inhibition of invadopodia formation. To elucidate that, we have quantified the number of invadopodia in 143B cells treated for the indicated time with AA/B-GP (**Fig. 8A**). Invadopodia in 143B cells were immunostained against cortactin and gelsolin and examined. **Figure 8A** illustrates that upon sustained treatment with AA/B-GP, the number of invadopodia formed by 143B cells (app. 3 per cell) was more than 4 times lower than under control conditions (13 per cell). The obtained data suggest that invadopodia formation in 143B osteolytic osteosarcoma cells *in* *vitro* is significantly disrupt by prolonged AA/B-GP stimulation. To additionally confirm the specificity of the above phenomenon, we performed gelatin degradation assay for control and AA/B-GP treated 143B cells (**Fig. 8B**). We observed normalized gelatin degradation per total area of cells to be 19.3±1.5% in control probes and 3.3±0.5% in AA/B-GP treated. Altogether, treatment of 143B cells with stimulators of mineralization limits proteolytic activity of invadopodia through an unknown mechanism.

## Discussion

Osteosarcoma is an extremely heterogeneous bone cancer. This heterogeneity has a profound impact on the effectiveness of the therapy [1]–[3], [15]. The results presented in this paper provide a detailed characteristic of two different phenotypes of osteosarcoma (osteoblastic and osteolytic) not only in terms of their mineralizing abilities but also cancerogenic potential. To our knowledge, this is the first laboratory study that evaluates the effect of AA/B-GP on migration and invasiveness of osteosarcoma cells of human origin, the key processes prerequisite for cancer metastasis.

First of all, comparison of the cellular level of osteogenic markers (TNAP [32], BMP-2 [33], CaSR [34]) and activity of TNAP confirmed Saos-2 cells to be competent to mineralization (osteoblastic), in contrast to 143B cells. The 143B cells tested here have an osteolytic phenotype and we proved that they did not initiate ECM mineralization upon a prolonged 7 day treatment with AA/B-GP. The inverse correlation of TNAP level and osteosarcoma cells proliferation was previously suggested [12], [21], [35] In our study the flow cytometry analysis further proved 143B cells to be highly proliferative contrary to mineralizing Saos-2 cells.

Here we confirmed that treatment with AA/B-GP stimulates osteoblastic Saos-2 cells to mineralization, as it was reported earlier [13], [14], [25], [26]. In agreement with [36], we observed that early mineralization of Saos-2 cells was accompanied by a decreased proliferation rate and cell cycle arrest in G0/G1. It has been recently proven that treatment of OS cells with other stimulators of mineralization, such as calcitriol (1,25-dihydroxyvitamin D_3_) [17], [18] or inorganic phosphate [37] results in growth inhibition. Moreover we observed that decreased rate of proliferation upon AA/B-GP treatment is accompanied by apoptosis in Saos-2 cells. Our results are consistent with the latest knowledge on cellular mineralization which points to apoptosis as one of the main mechanisms [38]–[42]. Additionally, we highlight the new observation that the early mineralization phase is accompanied by early apoptosis. The proapoptotic effect of AA/BGP in osteoblast-like Saos-2 cells was manifested not only by an increase in annexin V-PE but also by activation of caspases. Our results confirmed that apoptosis of mineralizing osteoblast-like Saos-2 cells might be partially related to caspase activation as it was previously suggested by others [43], [44]. Overall, the data indicated that apoptosis contributed to AA/BGP-mediated Saos-2 cell mineralization. In contrast to Saos-2 cells we evidenced that growth and viability of 143B was fully preserved upon AA/B-GP treatment. This observation further exposes the direct dependency of mineralization on cell apoptosis.

Clinical studies show that the more differentiated OS cells are, the less aggressive their phenotype is [10], [45]. Moreover, the latest research by [46], [47] revealed suppressory effect of calcitriol on prostate and breast cancer cells migration and invasiveness. This prompted us to investigate the influence of AA/BGP on OS cells spreading. Hence, we decided to monitor in detail the invasiveness of 143B in the presence of AA/B-GP. Different biological parameters may influence *in* *vitro* cell invasiveness, such as: (1) the growth rate, (2) the extent of cell migration, (3) adhesiveness to ECM and (4) ECM degradation facilitated by invadopodia formation [48], [49]. After testing the migration and invasiveness of our model cell lines we concluded that osteoblastic Saos-2 cells were in general non-invasive in contrast to osteolytic 143B.

We observed by time-lapse microscopy a decrease in migration of 143B and Saos-2 cells following treatment with AA/B-GP. Reduced migration of Saos-2 cells could be an effect of increased cell death. In the case of 143B cells the effect of AA/B-GP on migration is not due to affected cell viability. Our next observation revealed perturbation of 143B short-term adhesiveness to collagen type I in the presence of AA/B-GP. One of the possible explanations might be that AA/B-GP influences the integrin signaling pathways needed for proper cell adhesion [50]. Both adhesiveness and invasiveness are functionally linked in structures formed by invasive cancer cells called invadopodia [5]–[8]. Using invadopodia formation and matrix degradation assays, we evidenced that 143B, unlike Saos-2 cells, formed functional invadopodia with high frequency per cell. The ability of 143B cells to form invadopodia has not been described so far.

Furthermore, we observed for the first time the disregulation of invadopodia formation in human 143B osteosarcoma cells upon treatment with stimulators of mineralization. Thus, on the basis of our results, it can be concluded that the inhibitory effect of AA/B-GP on the invasiveness of OS cells may be due to disturbance of actin remodeling necessary for invadopodia formation. Most strikingly, we found that in 143B cells AA/B-GP affects cortactin distribution (actin remodeling protein known as invadopodia marker). The cortactin recruitment is essential for invadopodia formation downstream from multiple signals [51]–[54]. Our results support the notion that cortactin gene overexpression in osteosarcoma correlates with increased aggressiveness and reduced survival as in [9]. On the basis of the obtained results we suppose that AA/B-GP affected initial signals that trigger the establishment of invadopodia, followed by targeted secretion of proteases for ECM degradation. However, an alternative mechanism by which AA/B-GP impaired formation of invadopodia by 143B cells, could be related to the observed changes in their adhesion ability.

Taken together, we are the first to reveal that stimulators of mineralization act as inhibitors of osteolytic osteosarcoma cell invasiveness *in* *vitro*. Certainly, our data open a new area of studies on signaling pathways involved in AA/B-GP effect on invadopodia. Future studies are needed to determine whether the effect of AA/B-GP is replicated *in* *vivo* and elucidate if AA/B-GP can be used as a potential adjuvant to conventional therapy in aggressive, osteolytic bone cancer.
